# Precise Oligomer Organization Enhanced Electrostatic Interactions for Efficient Cell Membrane Binding

**DOI:** 10.1021/acs.nanolett.5c00651

**Published:** 2025-05-16

**Authors:** Yuanyuan Zhao, Yiqian Luo, Yi Chai, Yintung Lam, Yongqing Gong, Ke Chen, Gang Lu, Gang Xia, Yun Chang, Menghao Yang, Yang Xu, John Haozhong Xin

**Affiliations:** † School of Fashion and Textiles, 26680 The Hong Kong Polytechnic University, Hong Kong 999077, China; ‡ School of Materials Science and Engineering, 12476 Tongji University, Shanghai 201804, China; § Department of Neurosurgery, Renji Hospital, School of Medicine, 71140 Shanghai Jiao Tong University, 200127, Shanghai, China; ∥ School of Energy and Environment, 53025 City University of Hong Kong, Hong Kong 999077, China; ⊥ Department of Biomedical Engineering, 26680 The Hong Kong Polytechnic University, Hong Kong 999077, China

**Keywords:** electrostatic interactions, mechano-bactericidal, RAFT polymerization, zinc oxide nanorods, antibacterial surfaces

## Abstract

Efficient binding of cell membranes onto nanomaterials is essential for biomedical applications such as diagnostics and cellular engineering. We find that fine control over oligomer orientation led to enhanced electrostatic interactions with the cell membrane and improved cell membrane capture. Specifically, we designed polycation oligomers incorporating positively charged imidazole heads and alkyl tails synthesized through the reversible addition–fragmentation chain transfer (RAFT) reaction. These oligomers spontaneously self-assemble through head-to-head π–π interactions, and their spatial arrangement markedly accelerates the interaction with negatively charged cell membranes. Experimental results indicate that these oriented oligomers produce a large decrease in the time required to kill bacteria compared to unmodified nanostructures (3 min versus 100 min). This is attributed to locally concentrated electrostatic attraction, which enhances the attraction between nanostructures and negatively charged cell surfaces. Our findings suggest that molecular orientation control could be a promising approach to enhancing interactions between biomaterials and live cells.

## Graphical abstract

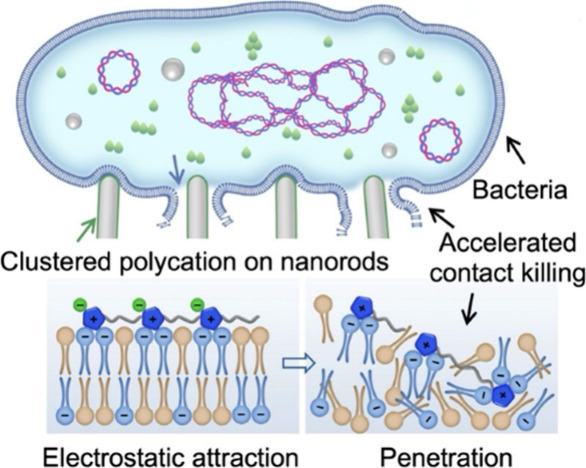

Cell membrane binding refers to the interaction of molecules or nanomaterials, such as proteins, lipids, or drugs, with the cell membrane, a selectively permeable barrier that surrounds and protects the cell.^1,2^ This binding is very essential for many functions that a cell executes, like signaling,^3,4^ transport,^5,6^ and the maintenance of cell integrity. It influences the way in which cells are adequately able to communicate with the environment surrounding them as well as among themselves, determining important functions such as nutrient uptake, immunity responses, and signal transduction.^7,8^ Another case is the adsorption of bacterial cell membranes and nanomaterials, which includes an interaction of nanomaterials with the outer membrane of bacteria.^9^ Nanoparticles can be designed to exhibit certain binding to bacterial cell membranes.^10–12^ The common thing is that they often take advantage of the many unique properties of bacterial surfaces,^13^ such as lipopolysaccharides in the case of Gram-negative bacteria^14,15^ and teichoic acids in the case of Gram-positive bacteria.^16,17^ Nanomaterials can target the membranes of bacterial cells, hence becoming good tools for combat antibiotic-resistant bacterial organisms,^18–20^ and develop new diagnostic techniques useful for bacterial infections.^21,22^ The manipulation of the binding of cell membranes is an important concept, giving rise to much further implications across most scientific and medical domains.

In this work, we show that physically clustered polycation oligomers can significantly amplify the interaction between the cell membrane and nanostructures. We synthesized carboxyl-terminated polycation oligomers using RAFT polymerization and applied them to zinc oxide nanorods (NR), which we selected as our model nanostructure due to their extensive use in research and straightforward direct comparisons. As shown in Figure 1a-c, the clustered polycation locally amplifies the electrostatic interactions between positively charged clusters and negatively charged bacteria.^23–25^ Additionally, the hydrophobic chains of the oligomers disrupt the cell phospholipid bilayer, leading to disorganization, disintegration, and intracellular cytoplasm leakage, ultimately causing cell wall destruction and lysis.^26–28^ We demonstrate that zinc oxide nanorods modified with polycation (PCaNR) exhibit exceptional antibacterial performance over bare NR (*E. coli* survival rate of 0% vs 86.5% after 3 min contact). Our work demonstrates that the contact killing properties of the nanomaterials can be significantly promoted by long-range electrostatic attraction combined with short-range van der Waals forces via well-aggregated polycations.

**Figure 1:**
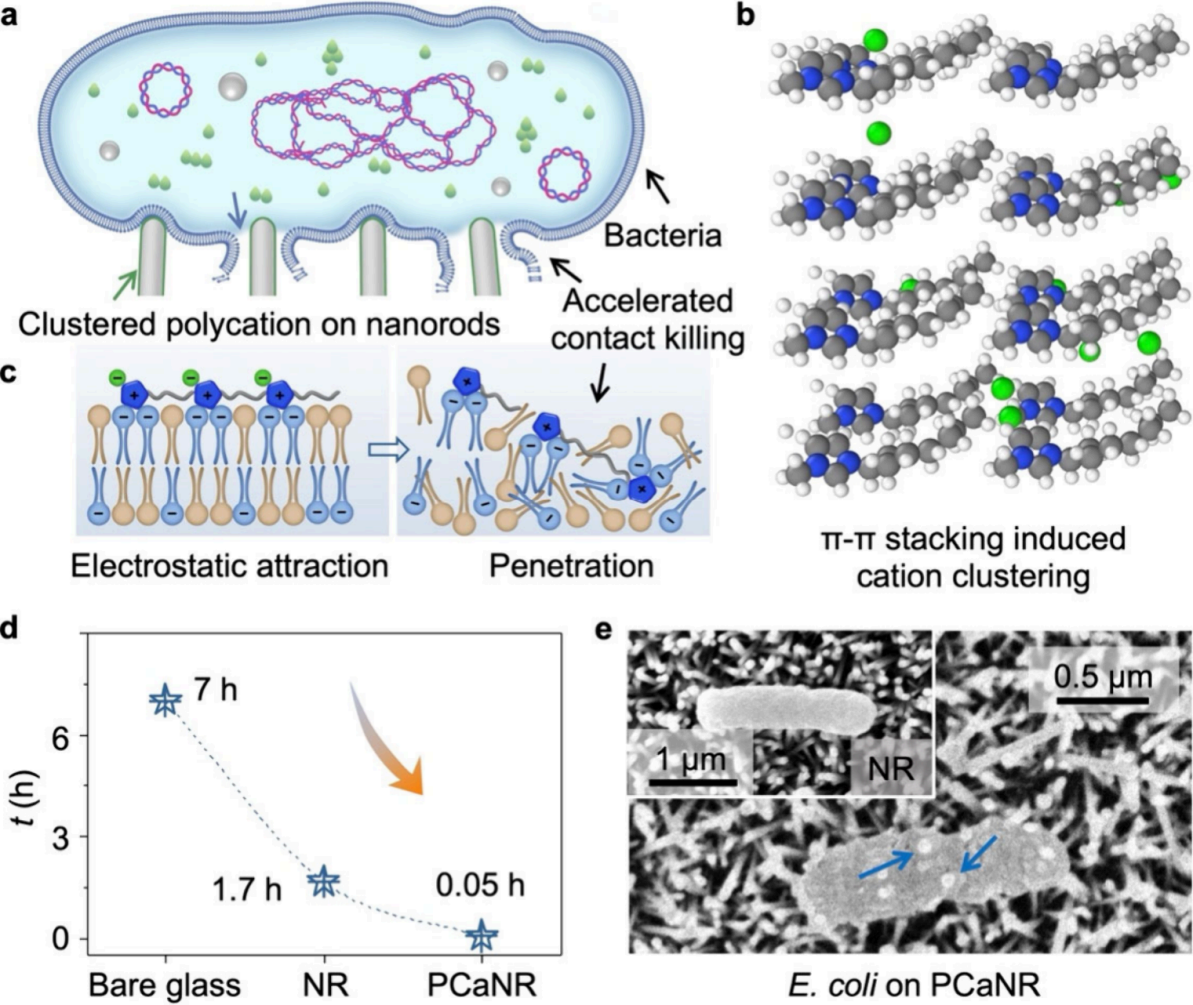
Mechanisms and performance of oligomer arrangement-enhanced binding of bacteria cell. (a) Cell membrane destruction by clustered polycation and the enhanced contact killing. (b) MD simulations of π–π stacking induce cation clustering. The balls of gray, blue, green, and white represent carbon, nitrogen, bromine, and hydrogen. (c) Mechanism of bacteria cell death resulting from PCa. (d) Time required for killing all airborne bacteria deposited on the three surfaces (bare glass, NR, and PCaNR). (e) SEM observations after airborne *E. coli* deposited on PCaNR, inset is the NR, showing disrupted and intact cell morphology. The error bars represent standard deviations, and *n* = 5 for each data point.

## Design and Fundamental Properties of PCaNR for Binding of Bacteria Cells

Biomimetic nanostructured surfaces have been confirmed exhibit strong mechano-bactericidal effects against many kinds of bacteria.^29–31^ *E. coli*, for example, is a Gram-negative bacterium and serves as a model organism for research. The typical structure of *E. coli* bacteria, as shown in Figure 1a, from outside to inside, comprises the cell wall, cell membrane, cytoplasm, nucleoid, and various intracellular structures. To enhance the mechano-bactericidal properties of nanostructures, we designed and synthesized a polycationic oligomer equipped with cationic imidazole rings and alkyl chains. In particular, the use of carboxyl modified RAFT agent enables the polymerization of antimicrobial cationic molecules and serves as surface engineering ligands for the assembly of cationic oligomers with zinc oxide nanorods.^32^ We hypothesize that the imidazole rings can undergo ordered stacking via π–π interactions, as illustrated in Figure 1b. This ordered molecular arrangement leads to a more concentrated distribution of cationic charges, which may exert a stronger attraction to the bacterial cell membrane, enhancing the antimicrobial effectiveness.^33–35^ Furthermore, the inclusion of alkyl chains is believed to speed up the compatibility of the nanostructure with the cell membrane.^36^ This compatibility facilitates a closer approach of the nanostructure to the bacterial cells, thus synergistically assisting in the disruption and killing of the bacteria, as depicted in Figure 1c. These features combined are expected to significantly improve the efficacy of the nanostructures in targeting and destroying bacterial pathogens, offering promising implications for the development of advanced antibacterial materials.

We used the zinc oxide nanorods because of the simple fabrication synthesis method and widespread applicability in mechano-bactericidal strategies.^22^ To test the antibacterial performance, we applied the bacteria containing aerosol on three kinds of surfaces, which were bare glass, NR, and PCaNR, and allowed contacting killing for a period until all bacteria dead.^37^ As shown in Figure 1d, the *E. coli* were killed on PCaNR in only 3 min, much faster than bare glass (7 h) and NR (100 min) surfaces. We then used the scanning electron microscope (SEM) to observe the morphology of the *E. coli* after contacting the three kinds of surfaces, in Figure 1e and Supplementary Figure 1. The images show the intact cell on bare glass and NR, while there is obvious cell collapse on the PCaNR. The preliminary antibacterial results, both the rapid elimination and deformation of bacteria, indicate the extraordinary efficiency of combining designed PCa and bioinspired nanorods in contact killing bacteria.

## Preparation and Characterization of PCaNR

We first prepared the PCa oligomer, and the synthesis process is illustrated in Supplementary Figures 2 and 3. Here, the RAFT polymerization method was employed to create a complex PCa architecture. PCa was composed of dual functional components: an anchoring group for the stable integration with NR, and cationic moieties that served as the antibacterial functional part. The initial phase entailed the fabrication of the RAFT agent, which the procedure for the synthesis of RAFT reagent was carried out.^38^ NR were then prepared using hydrothermal synthesis (Supplementary Figure 4). These nanorods underwent a surface modification with PCa, leveraging the terminal carboxylic group as the anchoring site, which obtained PCaNR (Supplementary Figure 5). In Figure 2a, the top-view of the PCaNR morphology shows that the diameter of PCaNR is about 82.3 nm. We performed Energy-dispersive X-ray (EDX) mapping to compare NR and PCaNR, as shown in Figure 2b and Supplementary Figure 6, which clearly highlights the differential content of nitrogen and bromine elements on the respective surfaces. X-ray photoelectron spectroscopy (XPS) analysis was employed to validate the modification, as depicted in Figure 2c and Supplementary Figure 7. Unlike NR, the XPS spectra for PCaNR show distinct peaks for the N 1s and Br 3d at binding energies of 401 and 68 eV, respectively. In Figure 2d, the water contact angle (CA) test reveals distinct wettability differences between the NR (approximately 13.1°) and PCaNR (about 151.8°). This contrast substantiates the efficiency of the modification process.^39^ PCa was successfully loaded on the NR as evidenced by Fourier Transform Infrared (FTIR) spectra (Figure 2e). Here, the absorption bands at 2920 cm^–1^, 1550 cm^–1^, and 1457 cm^–1^ can be ascribed to the stretching vibrations of C–H, C–C, and C=N bonds, respectively. The successful synthesis of PCa can be determined by ^1^H NMR spectroscopy analyses (Figure 2f). The signal peaks around 0.87 ppm were attributed to the end group CH_3_(H_c_) of the RAFT portion and CH_3_(H_d_) of PCa, while the signal peaks at 7.93 and 9.83 ppm can be attributed to the signal peaks of CH­(H_a_) and CH­(H_b_) on the imidazole.

**Figure 2:**
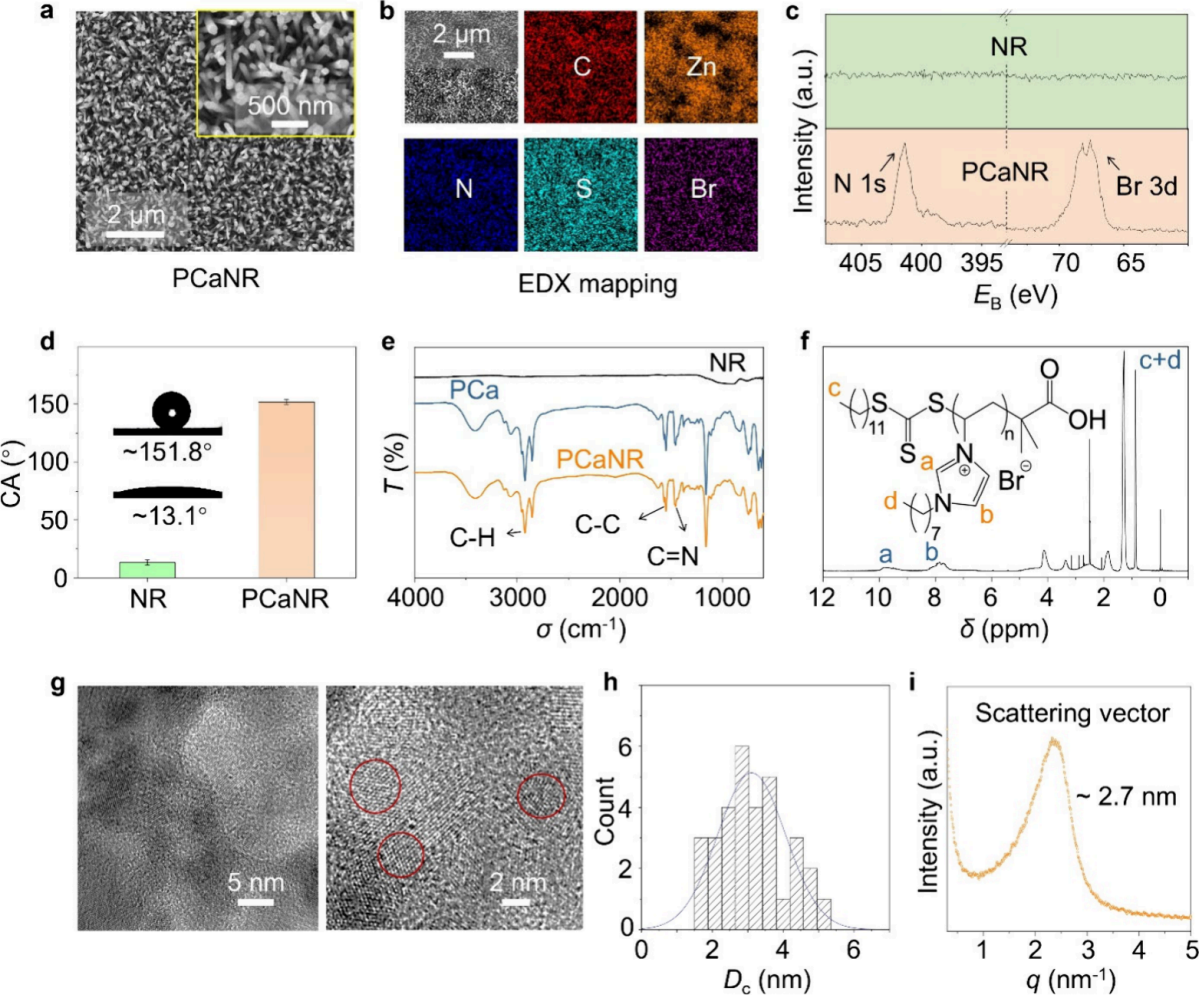
Characterization of PCaNR and the nanoclusters. (a) Scanning electron microscopy (SEM) images of PCaNR, inset is in higher magnification. (b) EDX mapping of the PCaNR. (c) High-resolution XPS spectra (N and Br elements) of NR (top) and PCaNR (bottom). (d) Water contact angle of NR and PCaNR, insets show the optical images with different wettability. (e) FTIR spectra of the NR, PCa, and PCaNR. (f) NMR spectroscopy of PCa. (g) HRTEM images of PCaNR. Left is TEM image at a lower magnification. Right is High-resolution TEM image, and the red circles mark the representative well-ordered nanoclusters. (h) Histogram of the nanocluster size distribution. (i) SAXS data of PCa. The error bars represent standard deviations and *n* = 5 for each data point.

To confirm our hypothesis regarding the nanoclusters via head π–π interactions, we then conducted HRTEM and small-angle X-ray scattering (SAXS).^40^ As shown in Supplementary Figure 8, the TEM image and related EDX mapping of different elements confirmed the PCa chemical composition. We can find the nanoclusters from Figure 2g, and the representative nanoclusters have been red circled. The size distribution is plotted in Figure 2h, with an average diameter of about 3.1 nm, which is well aligned with the SAXS results of ∼2.7 nm.

## Bacteria Contact Induced Binding and Killing

*E. coli*, a well-known bacterial model, was selected for assessing the antibacterial efficiency of PCaNR. The antibacterial assays were performed in the absence of light to eliminate the influence of reactive oxygen species. In order to investigate the mechanisms and dynamics of bacterial eradication, we utilized bare glass, NR, and PCaNR as comparative surfaces. We carried out the contact-killing test over various time intervals to evaluate the antibacterial capabilities through agar plate colony counting. We then quantified *E. coli’*s survival rate on the three surface types for different contact times (Figure 3a). Figure 3b distinctly illustrates the sharp decline in the contact-killing time of PCaNR (3 min), compared to bare glass (∼7 h) and NR (∼100 min), indicating PCaNR’s significantly enhanced bactericidal activity. We aerosolized an *E. coli* suspension onto the three types of surfaces and maintained contact for 3 min.

**Figure 3:**
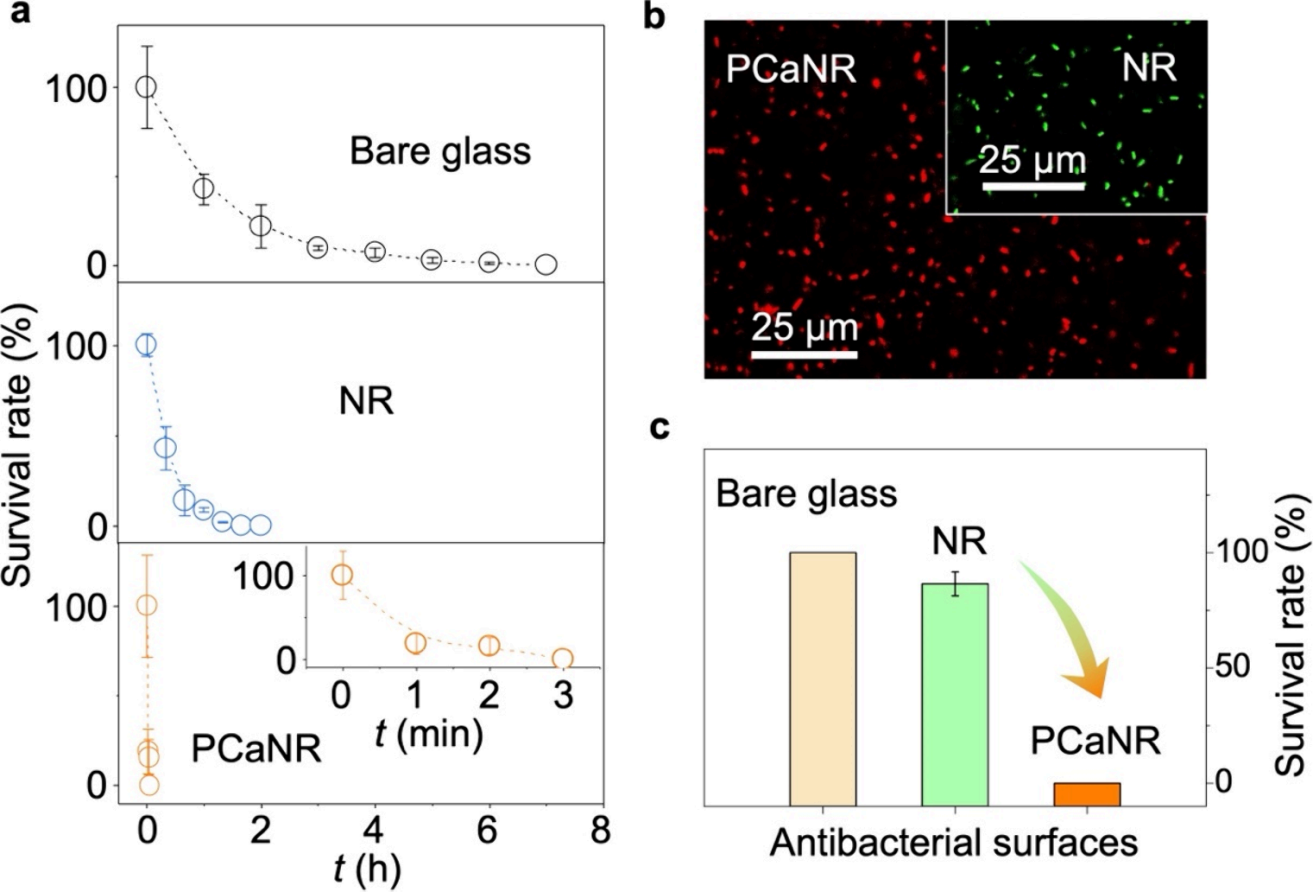
Bacteria contact-killing behaviors of PCaNR. (a) Time-dependent *E. coli* survival rate on bare glass, NR, PCaNR surfaces. (b) Representative fluorescent microscope imaging after airborne *E. coli* deposited on NR, PCaNR surfaces. (c) The bacteria survival rate after contact-killing for 3 min on three kinds of surfaces. The error bars represent standard deviations and *n* = 5 for each data point.

Fluorescent microscopy imaging was further performed, as shown in Figure 3b and Supplementary Figure 9, with green fluorescence indicating live *E. coli* and red indicating dead bacteria. The marked contrast in the live/dead ratio (Figure 3c) underscores the exceptional bactericidal performance of PCaNR. To confirm the extraordinary microbicidal performance derived from the combination of PCa and nanostructure, we prepared bare glass and PCa modified glass (PCa@glass). Supplementary Figure 10a and b demonstrate the wettability difference between bare glass (∼32°) and PCa@glass (∼93°). The XPS spectra (Supplementary Figure 10c) compare the element composition of two surfaces, the N 1s and Br 3d peaking at 401 and 68 eV for PCa@glass, showing the successful preparation. In Supplementary Figure 10d, demonstrating the antibacterial behaviors, we can determine that the survival rates of bare glass and PCa@glass remain at a relatively high level (greater than 75%) after contact with bacteria. Considering the bactericidal actions of PCa@glass and PCaNR, we believe the combination of nanoclustered PCa and NR produces the extraordinary antimicrobial results.

We further demonstrated the detailed antibacterial effectiveness of the different PCaNR. We prepared three different types of PCaNR1–3 with a solution of PCa in methanol at 0.001, 0.1, and 10 mol/L, respectively. The water contact angles, as displayed in Figure 4a and Supplementary Figure 11, were measured. With an increasing concentration, the surface properties changed from a hydrophilic to a hydrophobic, and to a superhydrophobic surface with contact angles of up to 150°. Furthermore, an EDX mapping analysis was conducted to determine the bromine element composition across the different surfaces, with results presented in Figure 4b. This analysis highlighted the compositional diversity among the three types of surfaces. The antibacterial assays, as depicted in Figure 4c, d, and Supplementary Figure 12, revealed a pronounced reduction in bacterial killing-contact time. Compared to the 1.7 h observed for NR (Figure 3b), the modified surfaces demonstrated significantly decreased bacterial survival times of 3 min, 9 min, and 1 h for PCaNR1–3, respectively. This reveals the improved antibacterial performance with PCaNR surfaces. Moreover, the bactericidal performances of PCaNR demonstrate suitability for a broad range of environmental humidity conditions, as displayed in Figure 4e, in strong contrast to NR, which is drastically influenced by the increasing RH. The above results indicate that nanoclustered PCa plays an important role in the enhancing mechano-bactericidal actions.

**Figure 4:**
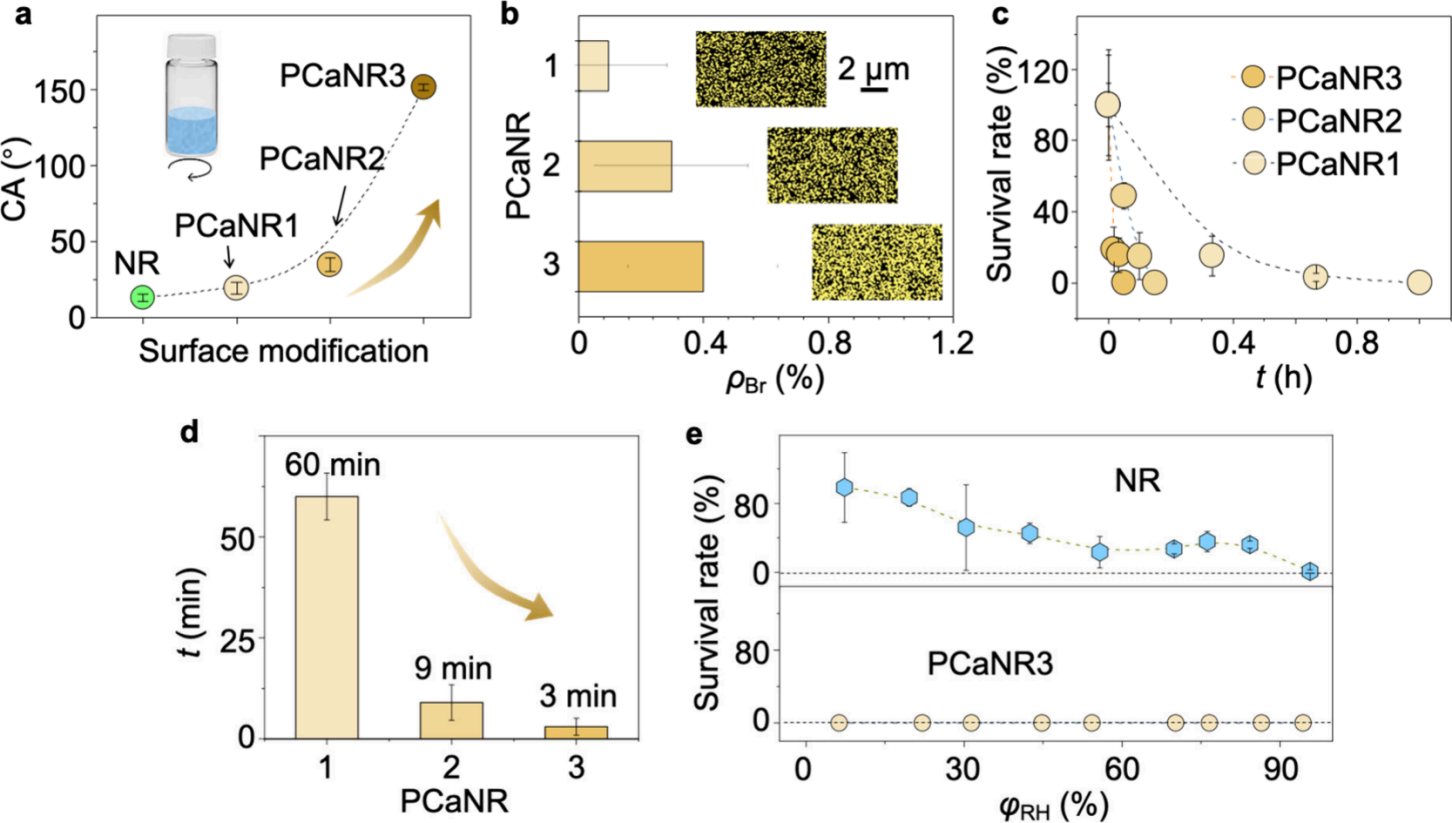
Detailed bacterial killing performance of PCaNR. (a) Water contact angle of NR and PCaNR1–3. (b) Br element concentration and EDS mapping of PCaNR1–3. (c) Time-dependent *E. coli* survival rate on PCaNR1–3 surfaces. (d) Time required for killing all airborne bacteria deposited on the three surfaces. (e) *E. coli* survival rate of NR and PCaNR under a series of humidity conditions. The error bars represent standard deviations and *n* = 5 for each data point.

## Molecular Dynamics (MD) Simulation for Molecular Orientation Enhanced Bacteria Binding and Killing

To further figure out how molecular arrangements provoke antibacterial effects, we simulated the arrangement of this cation using all-atom MD simulations. The results (Figure 5a-b and Supplementary Figure 13) show that pyridine π–π stacking interaction allows clean packing and ordered disposition of cations. From Figure 5c-d and Supplementary Figure 14, when we had ordered these well-packed molecules (PCa-2) on the surface of a lipid bilayer, it was observed that the polycations under alignment interacted more closely, moving toward one another at a faster rate than the unaligned cations (PCa-1), as shown in Figure 5c and d. Specifically, when these PCa-2 molecules were densely packed (Figure 5e), the distances were 6.55 and 3.68 Å after equilibrium, respectively. This is due to the optimization of electrostatic interactions by the orderly arrangement of charges, which promotes interaction between the bacteria cell membrane and PCaNR. These results suggest that by orderly molecular orientation, the interaction between bacteria and antibacterial surfaces can be locally enhanced, promoting antibacterial effects.

**Figure 5:**
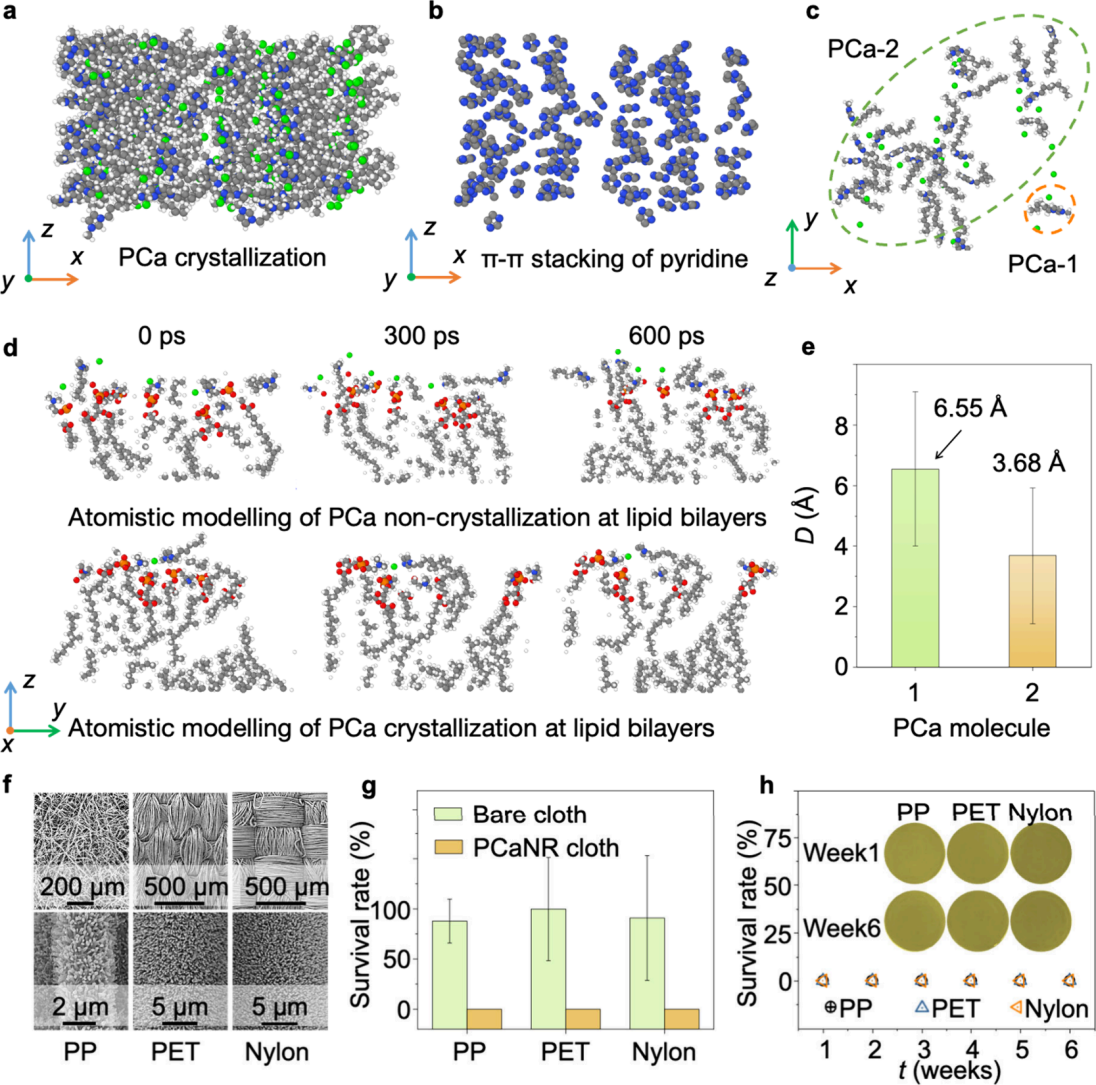
Atomistic arrangements of lipid bilayers binding with PCa and application of PCaNR for bactericidal fabrics. (a) Crystallized configuration of PCa. (b) Structural relaxation led to π–π stacking of pyridine. (c) Representative configurations of PCa-1 (noncrystallized) and PCa-2 (crystallized). (d) Time-dependent noncrystallization and crystallization of PCa within lipid bilayers. (e) Averaged interatomic distance between lipid bilayers and PCa molecules, with error bars indicating standard deviations for each data point. (f) SEM images of PP, PET, and Nylon cloth modified with PCaNR. (g) Survival rate on bare and PCaNR modified PP, PET, and Nylon cloth. (h) Long-term antibacterial tests for PCaNR based cloth. The error bars represent standard deviations and *n* = 5 for each data point.

## Bioprotection Application of PCaNR

Based on our results, we successfully proved the possibility of using PCaNR as a biocidal surface layer for bioprotection applications. We functionalized three types of fabric for this purpose: melt-blown polypropylene (PP), poly­(ethylene terephthalate) (PET), and Nylon. Figure 5f shows the altered morphology of the fabric fibers after modification. The wettability of the fabrics was tested by recording the contact angles of water with the fabrics before and after modification, which are measured and shown in Supplementary Figure 15. The results indicate that PP retains its superhydrophobicity, whereas PET and Nylon become hydrophilic to superhydrophobic, with water contact angles exceeding 150°, thus indirectly confirming successful modification. In the antimicrobial efficiency tests, *E. coli*-laden aerosols were applied to the various fabric surfaces for a contact period of 3 min. The resulting survival rates drastically fell from 87% for untreated PP, 90% for untreated PET, and 89% for untreated Nylon to complete eradication of the bacteria on the treated fabrics, as evidenced in Figure 5g and Supplementary Figure 16. To assess the durability of the antibacterial effect, we conducted longitudinal antimicrobial tests. The results, illustrated in Figure 5h and Supplementary Figure 17, demonstrated zero survival rate for bacteria on the treated fabrics for a duration of 6 weeks, indicating the outstanding stability of the antibacterial properties. Based on the above results of the systematic bactericidal tests, we have preliminarily proven the application of PCaNR in the field of bioprotection.

These findings are consistent with our hypothesis that clustering polycations locally can enhance the electrostatic attraction between bacterial cells and nanostructures and also increase van der Waals forces to accelerate cell membrane penetration. Practical testing showed the disinfection efficiency of PCaNR under variable humidity when used as a means of imparting superhydrophobic properties to textiles. This proves the feasibility of using enhanced polycation clustering for the binding-induced sterilization of airborne pathogens in practice.

At the same time, our method of enhancing interactions between cell membranes and solid surfaces through polycation clustering might find multiple applications, ranging from medical device coatings to pharmaceutical ones. For instance, polycation clustering can enhance the transport of the latter through the cellular membrane, which ensures greater efficiency of drug delivery systems with improved selectivity and cell penetration. In further work, it is also proposed to develop polymers with better clustering capability or possibly responsive to specific stimuli with the aim of enhancing multifunctional performance in terms of mechano-bactericidal actions.

## Supplementary Material



## Notes

Detailed information on the relevant preparations, characterizations, measurements, and performance. (PDF)
