# Supplementary material for: Precise Oligomer Organization Enhanced Electrostatic Interactions for Efficient Cell Membrane Binding
Source: Nano Lett. 2025 May 16;25(21):8488–94. doi: 10.1021/acs.nanolett.5c00651 (PMC12123663; doi:10.1021/acs.nanolett.5c00651)
Supplement: Supplementary file 1 [file nl5c00651_si_001.pdf]

Supplementary information

Precise oligomer organization enhanced electrostatic interactions for efficient cell membrane binding

Yuanyuan Zhao[1#], Yiqian Luo[2#], Yi Chai[3#], Yintung Lam[1], Yongqing Gong[2], Ke Chen[2], Gang Lu[4], Gang Xia[1], Yun Chang[5], Menghao Yang[2], Yang Xu[2*], John Haozhong Xin[1*]

[1]School of Fashion and Textiles, The Hong Kong Polytechnic University, Hong Kong 999077, China

[2]School of Materials Science and Engineering, Tongji University, Shanghai 201804, China

[3]Department of Neurosurgery, Renji Hospital, School of Medicine, Shanghai Jiao Tong University 200127, Shanghai, China

[4]School of Energy and Environment, City University of Hong Kong, Hong Kong 999077, China

[5]Department of Biomedical Engineering, The Hong Kong Polytechnic University, Hong Kong 999077, China

Content

Supplementary Fig. 1 SEM observation after airborne *E. coli* deposited on bare glass.
Supplementary Fig. 2 Schematic illustration of the fabrication of RAFT agent and PCa.
Supplementary Fig. 3 The demonstration of PCa molecular structure (ball-and-stick model).
Supplementary Fig. 4 SEM images of NR.
Supplementary Fig. 5 The antibacterial behaviors causing from the combination of nanostructure and PCa.
Supplementary Fig. 6 EDX mapping of NR.
Supplementary Fig. 7 XPS spectra of NR and PCaNR confirming successful loading of the N and Br elements.
Supplementary Fig. 8 TEM image and EDX mapping of S, Br, C, N, O elements.
Supplementary Fig. 9 Representative fluorescent microscope imaging after airborne *E. coli* deposited on bare glass.
Supplementary Fig. 10 Excluding the only role of PCa in the microbicidal performance of PCaNR.
Supplementary Fig. 11 Representative optical images of water contact angle on NR and PCaNR1-3.
Supplementary Fig. 12 Optical images of representative agar plates of sprayed bioaerosol onto PCaNR1-3 for different time periods.
Supplementary Fig. 13 Major ionic packings in the crystallization of imidazolium - based molecules.
Supplementary Fig. 14 MD atomistic simulation configuration at 300 ps.
Supplementary Fig. 15 Representative optical images of water contact angle on various fabrics.
Supplementary Fig. 16 Representative optical images of 3-minute antibacterial assay results of bare cloth and PCaNR based cloth.
Supplementary Fig. 17 Representative optical images of long-term antibacterial assay results for PCaNR based cloth.

Methods

Materials. 2-mercaptoethanol (Adamas), Tripotassium phosphate (K_3PO_4, Sinopharm Chemical Reagent Co.,Ltd), Carbon disulfide (CS_2, Adamas), Benzyl bromide (Adamas), Azobisisobutyronitrile (AIBN, Sinopharm Chemical Reagent Co.,Ltd), 1-octyl-3-vinylimidazolium bromide (Zhuoyan Chemical Technology Co., Ltd.), deuterated DMSO (Adamas), Zinc acetate (Sinopharm Chemical Reagent Co.,Ltd), Ethanolamine (AR, 99%, Aladdin), 2-Methoxyethanol (AR, Aladdin), Zinc nitrate hexahydrate (98%, Alfa Aesar), Hexamethylenetetramine (99%, International laboratory USA), Glutaraldehyde (Alfa Aesar, 50% aq. soln. 50% aq), Phosphate-buffered Saline (PBS, pH 7.4, Sigma), nutrient agar (HKM 022021), nutrient broth (HKM 022010), SYTO 9 (Thermo Fisher Scientific, 3.34 mM in dimethylsulfoxide), Prodium iodide (PI, Thermo Fisher Scientific, 20 mM in dimethylsulfoxide), Lithium bromide (LiBr, 99%, Aladdin), Potassium acetate (CH_3COOK, 99%, Aladdin), Magnesium chloride ($MgCl_2$, 99%, Aladdin), Potassium carbonate (K_2CO_3, 99%, Aladdin), Magnesium nitrate hexahydrate ($Mg(NO_3)_2 \cdot 6\ H_2O$, 99%, Sigma-Aldrich), Potassium iodide (KI, 99%, Aladdin), Sodium chloride (NaCl, 99%, Aladdin), Potassium chloride (KCl, 99%, Aladdin), Ethanol (AR, UNI-CHEM), Acetone (Sinopharm Chemical Reagent Co.,Ltd), Methanol (Sinopharm Chemical Reagent Co.,Ltd), Deionized water (Thermo Scientific), Dimethylformamide (Sinopharm Chemical Reagent Co.,Ltd).

Synthesis of RAFT reagent and PCa. Add C2-mercaptoethanol, K_3PO_4 (1.35 g, 1.26 mmol), and acetone (10.00 mL) to a round-bottomed flask and stir the mixture for 10 minutes. Then, CS_2 (1.46 g, 19.25 mmol) was added to the above mixture, and after the resulting yellow solution was stirred for 10 min, benzyl bromide (1.10 g, 6.42 mmol) was added to the reaction. Stir for another 30 minutes to filter out the solid, remove the solvent, and remove from the organic filtrate under reduced pressure. The initial product was further purified by column chromatography to obtain a yellow oily liquid (RAFT agent). Take an appropriate amount of 1-octyl-3-vinylimidazolium bromide monomer, RAFT agent, and AIBN in the ratio [monomer: RAFT Agent: AIBN = 100:2:1]. Sequentially introduce them into a 25 mL pressure-resistant tube and dissolve with 5 mL DMF under stirring. The reaction vessel was degassed by three freeze–pump–thaw cycles. After returning to room temperature, it was stirred for 20 h at 60 °C, and quenched in liquid nitrogen. The polymer was precipitated from acetone into ethanol three times to obtain a light-yellow solid product. The solid product was dried overnight in a vacuum oven at room temperature and stored. The product was dissolved in deuterated DMSO for NMR analysis.

Fabrication of NR and PCaNR. We applied hydrothermal synthesis to prepare NR and PCaNR. We firstly dissolved zinc acetate and ethanolamine in glycol methyl ether. This mixture was subjected to ultrasonic treatment until it became a homogenous solution without any precipitates and this was noted ZnO seed solution. Next, we dissolved zinc nitrate hexahydrate and hexamethylenetetramine in distilled water, and this was taken as the growth solution. The solution that was ultrasonic treated became transparent, and this growth solution was transparent, therefore, noted ZnO growth solution. We cleaned the glass slides by immersing them in distilled water and ethanol stepwise, each for five minutes, and after each, we sonicated and cleaned it. We then deposited a few drops of ZnO seed solution on the various cleaned slide and spin-coated it for a more comfortable and uniform spreading of the solution. We then incubated the slide on a hot plate that had been preheated to 230 C for 15 minutes. Following this, the slides were immersed in the ZnO growth solution and heated at 85°C for 9 hours for hydrothermal synthesis. After the growth phase, the prepared NR were rinsed with deionized water and allowed to dry. The NR were then subjected to a further process where it was submerged in PCa solutions of methanol for 20 minutes, rinsed with methanol, and left to dry, which yielded the PCaNR. For fabric substrates, a similar method was employed, with the modification that the fabric was immersed in the ZnO seed solution for 30 minutes and then incubated on a hot plate at 100°C for 15 minutes, reflecting the only variation from the glass substrate process.

Characterization. SEM (Tescan MAIA3) was utilised to observe the morphology. EDX was employed to map the element composition. FT-IR spectra meter (Thermo Nicolet iS50) were used to analyse NR, PCa, and PCaNR. NMR spectroscopy (Bruker Avance III 600 MHz) was applied to test PCa with functional groups. Water contact angle was tested via SDC-100Standard contact angle instrument. XPS (Thermo Scientific Nexsa) was used to identify the surface elemental composition. TEM (FEI Talos F200x) was utilized to observe the morphology of nanoclusters. SAXS (Anton Paar SAXSess mc2) was used to analyze the nanostructure of PCa.

TEM sample preparation. The PCaNR samples were initially synthesized on glass substrates. After completion of the surface modification, the coated nanorods were gently scraped off from the glass surface and transferred into an agate mortar. The collected material was ground manually for 10 minutes to break apart agglomerates and reduce particle size. The ground

powder was then dispersed into 300 μL of methanol under mild stirring. After standing for 10 minutes, the upper clear suspension was carefully collected to minimize large debris. A drop of the supernatant were drop-cast onto a carbon-coated copper TEM grid, and the solvent was allowed to evaporate naturally at room temperature.

Antimicrobial assay. An antimicrobial test was conducted using *E. coli* strain CMCC 44102. An *E. coli* colony on the surface of an agar plate was picked into 10 mL of nutrient broth and was cultured at 37°C for 24 hours. The suspension was washed with PBS, followed by centrifugation with concentration (8000×g for 15 min, Thermo Fisher Scientific Centrifuge Pico 17). The resulting bacterial precipitation was re-dispersed in a PBS solution. The obtained bacterial concentration was verified by performing serial dilutions and plating using the Colony Forming Unit (CFU) counts. For the antimicrobial assay, 3 mL of the bacterial suspension with a density of 10^7 CFU/mL was added using a compression atomizer (XBK, MCN-S600MF) to create *E. coli*-containing aerosol[1, 2]. It was sprayed onto the sample surface from a 25 cm distance for a duration of 30 seconds, contacting for a certain period. The bacteria were washed with ultrasound and plated on agar plates. Specifically, the samples were immersed in 3 mL PBS, and the surface-associated bacteria were released into the solution using mild sonication (40 kHz, 1 min). Subsequently, the bacterial suspension was transferred and uniformly plated onto agar dishes for colony enumeration. Then, the plates were incubated at 37 °C for 24 h for antibacterial efficacy evaluation. For the SEM study, the bacteria from the samples were removed by ultrasound and three times washed with PBS. The bacteria were fixed in a 2% glutaraldehyde solution in PBS and incubated at 4 °C overnight. The cells were then washed several times with deionized water. The dehydration of the bacterial samples was achieved through a graded series of ethanol/water mixtures, with ethanol concentrations at 30, 50, 70, 90, 95, and 100%, an hour for each concentration. The final preparation step involved coating the bacterial cells with gold to make them suitable for SEM analysis. For live/dead fluorescence assessment, the bacterial suspension was stained with SYTO 9 green-fluorescent dye and propidium iodide (PI) red-fluorescent dye for 20 minutes. After staining, the bacteria were washed with PBS and resuspended in it. A fluorescence microscope (Nikon Eclipse Ti2) was used to observe and document the fluorescence. Antibacterial tests were run under different controlled humidity levels with the use of saturated aqueous solutions method[3].

All-atom MD simulation. The procedures used here to prepare the lipid bilayer molecules were the same as described previously[4]. We incorporated 25 PCa molecules into the lipid

bilayers. The initial dimensions of the membrane models were 54 nm × 54 nm × 102 nm, comprising a total of 13,334 atoms for the lipid bilayers. Subsequently, we conducted all-atom MD simulations of PCa molecules within lipid bilayers using the NAMD software, employing the CHARMM36 lipid force field[5] and the modified TIP3P water model[6]. Periodic boundary conditions were applied in the X, Y, and Z directions of the membrane systems.

In these MD simulations, we kept the membrane systems in a tetragonal unit cell configuration to keep a constant size in the X and Y directions over the membrane plane and still allowed free expansion/contraction in the Z direction in the NPT ensemble. We used Langevin dynamics to keep the temperature at 298 K for each membrane system. We used the Langevin-piston algorithm to keep the pressure at a constant 1.01325 bar, which corresponds to the atmospheric pressure at sea level. For efficient calculation of long-range electrostatic interactions, we employed the particle-mesh Ewald (PME) method. The grid sizes utilized were $60 \times 60 \times 10^5$ for the membrane systems. It was simulated for 10 ns after the equilibrium period of 100 ps with a time step of 2 fs.

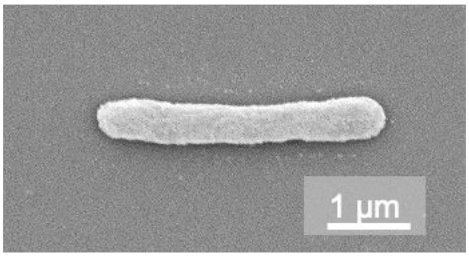

Supplementary Fig. 1 SEM observation after airborne *E. coli* deposited on bare glass.

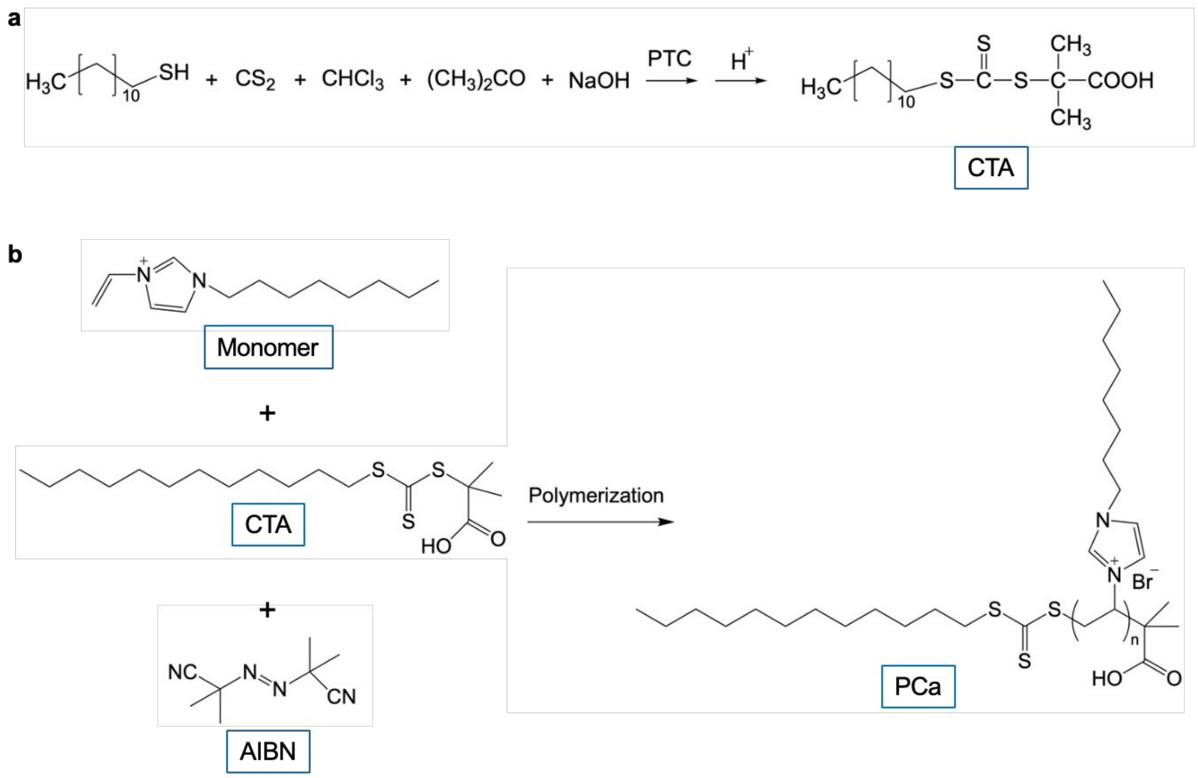

Supplementary Fig. 2 Schematic illustration of the fabrication of RAFT agent (a) and PCa (b).

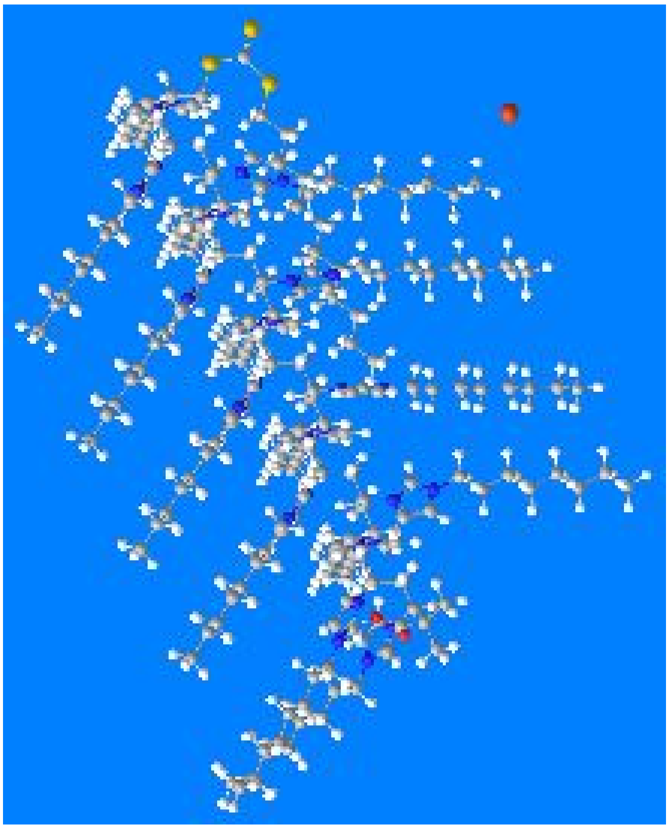

Supplementary Fig. 3 The demonstration of PCa molecular structure (ball-and-stick model).

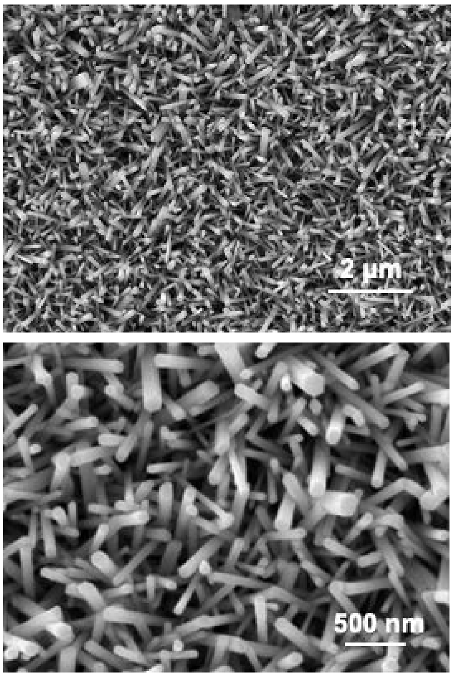

Supplementary Fig. 4 SEM images of NR.

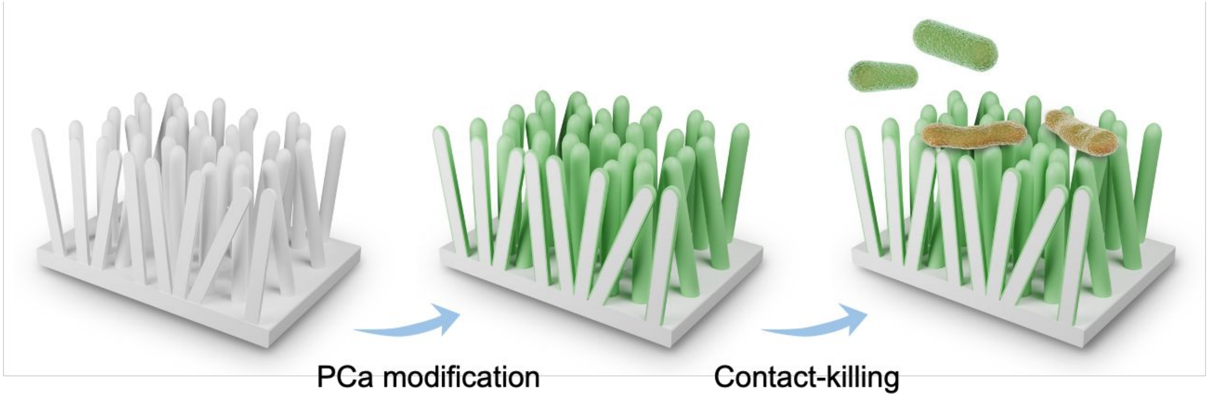

Supplementary Fig. 5 The antibacterial behaviors causing from the combination of nanostructure and PCa.

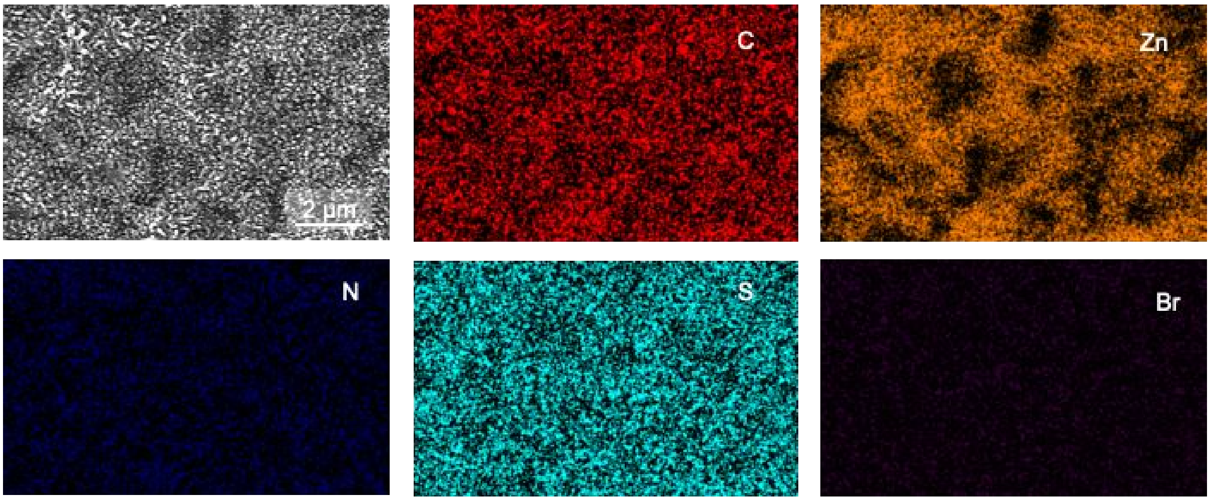

Supplementary Fig. 6 EDX mapping of NR.

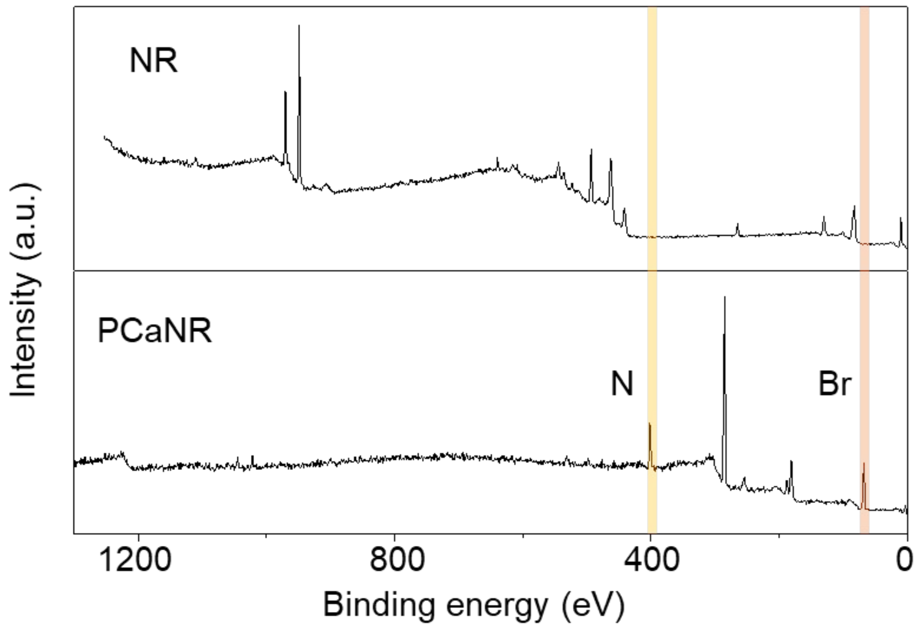

Supplementary Fig. 7 XPS spectra of NR and PCaNR confirming successful loading of the N and Br elements.

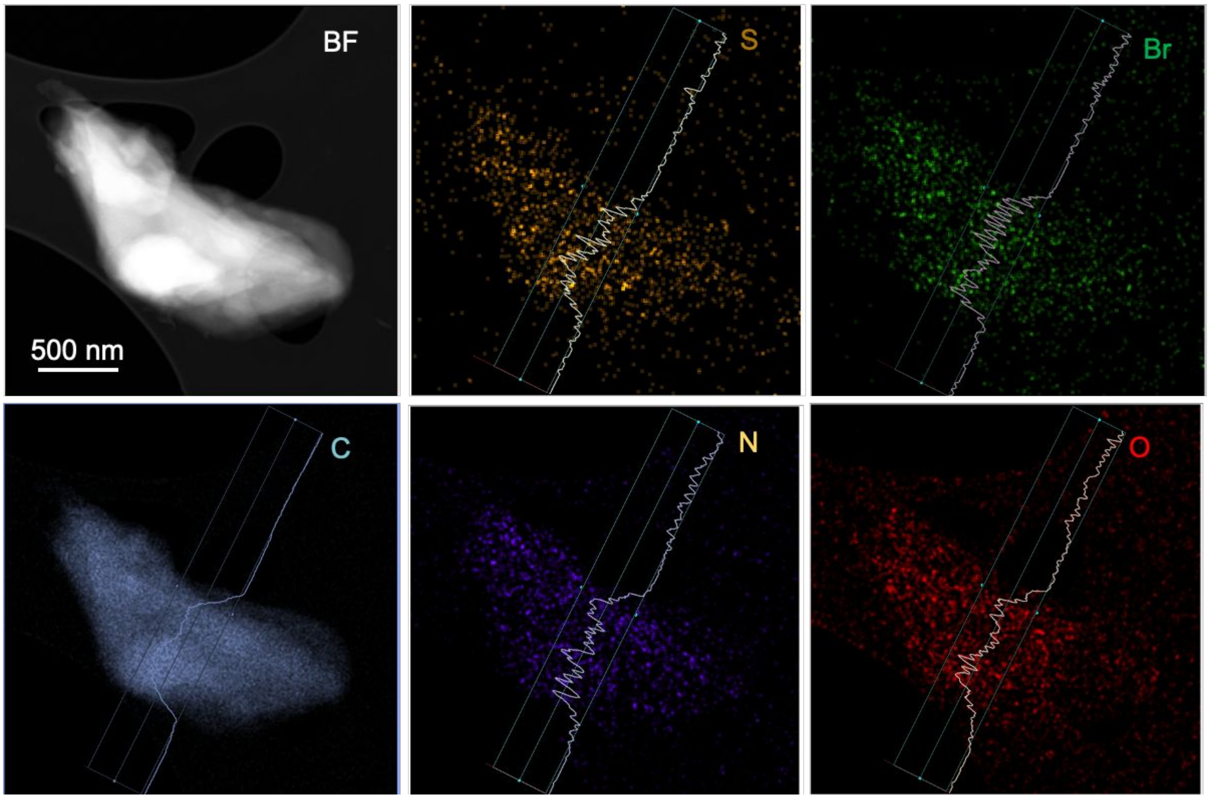

Supplementary Fig. 8 TEM image and EDX mapping of S, Br, C, N, O elements.

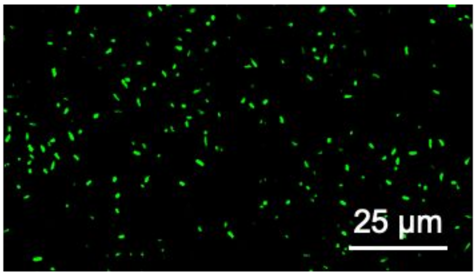

Supplementary Fig. 9 Representative fluorescent microscope imaging after airborne *E. coli* deposited on bare glass.

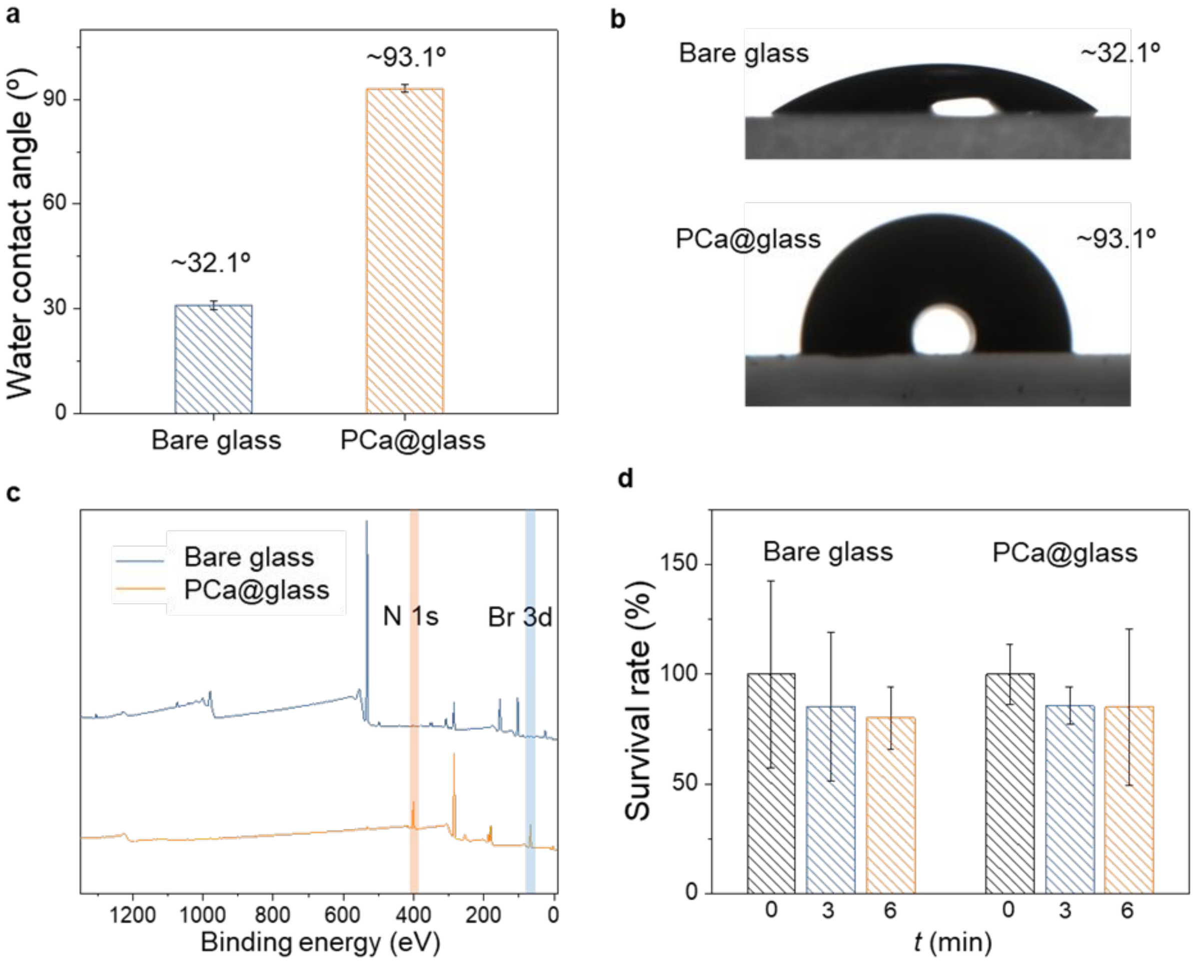

Supplementary Fig. 10 Excluding the only role of PCa in the microbicidal performance of PCaNR. a water contact angle of bare glass and PCa@glass. **b** representative optical images of bare glass and PCa@glass. **c** XPS spectra of bare glass and PCa@glass. **d** *E. coli* survival rate of bare glass and PCa@glass.

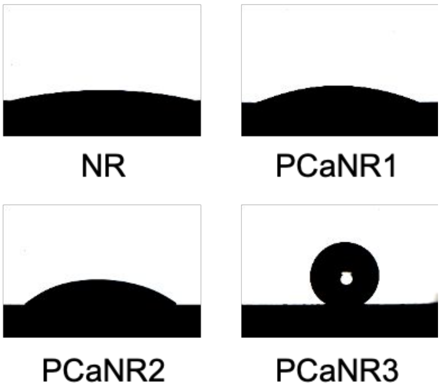

Supplementary Fig. 11 Representative optical images of water contact angle on NR and PCaNR1-3.

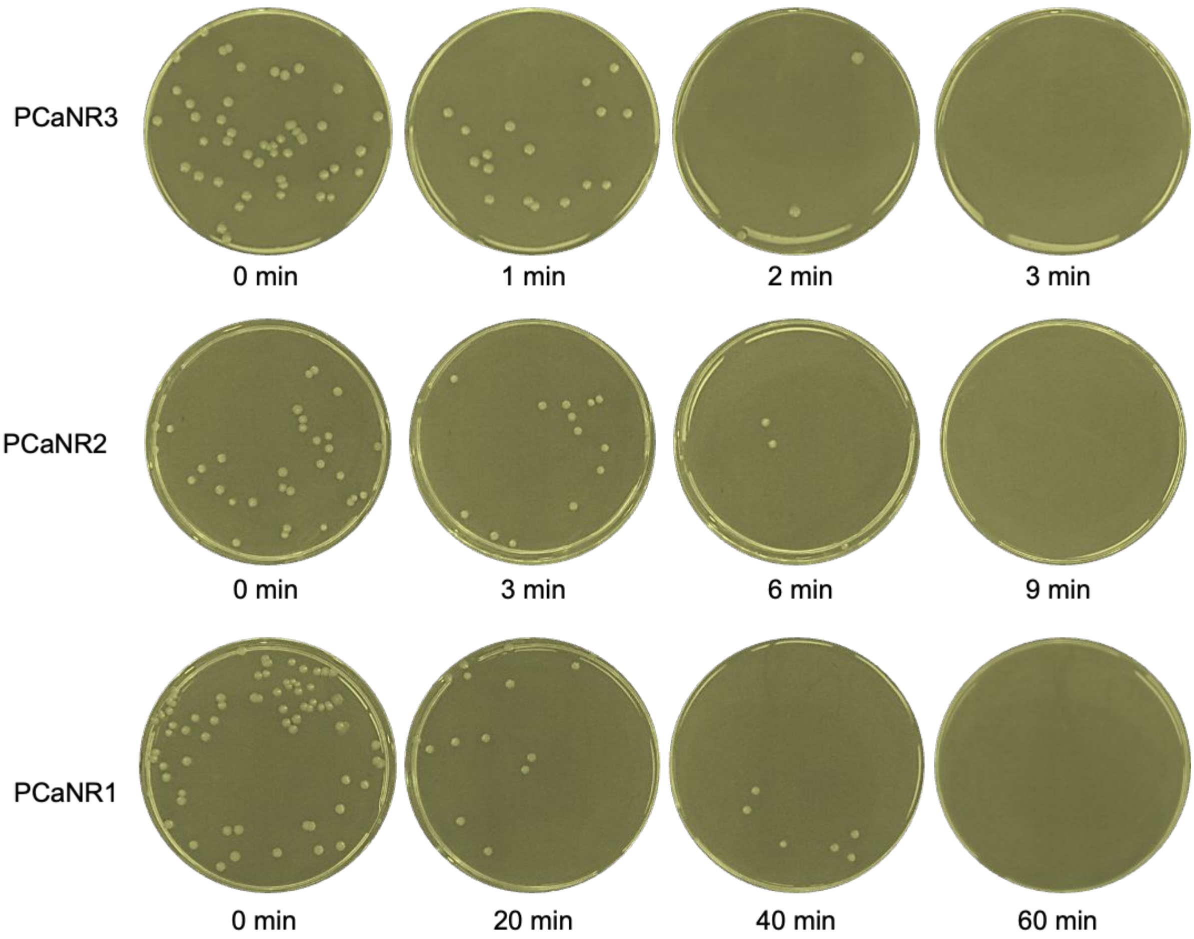

Supplementary Fig. 12 Optical images of representative agar plates of sprayed bioaerosol onto PCaNR1-3 for different time periods.

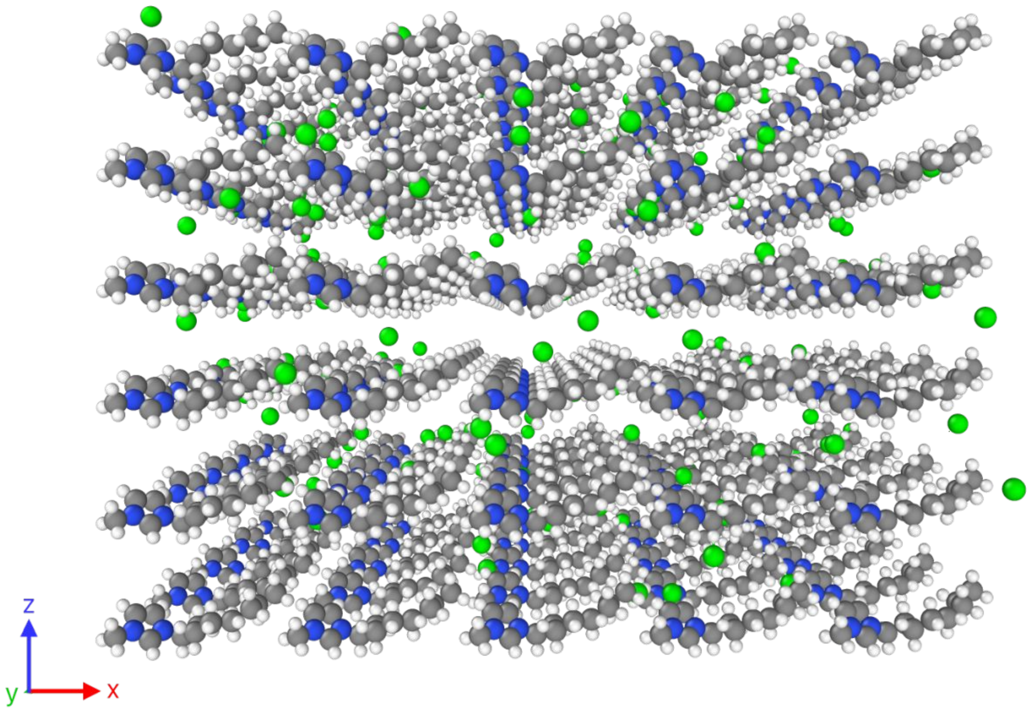

Supplementary Fig. 13 Major ionic packings in the crystallization of imidazolium - based molecules.

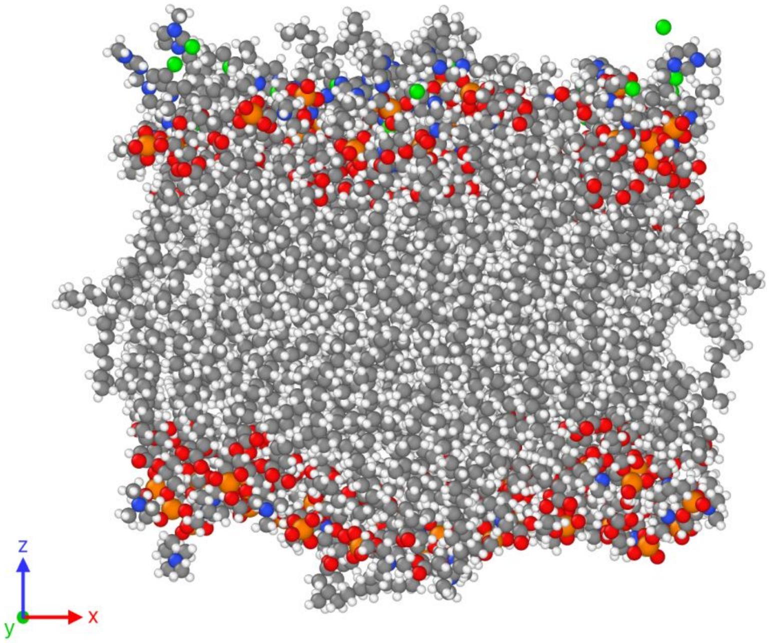

Supplementary Fig. 14 MD atomistic simulation configuration at 300 ps.

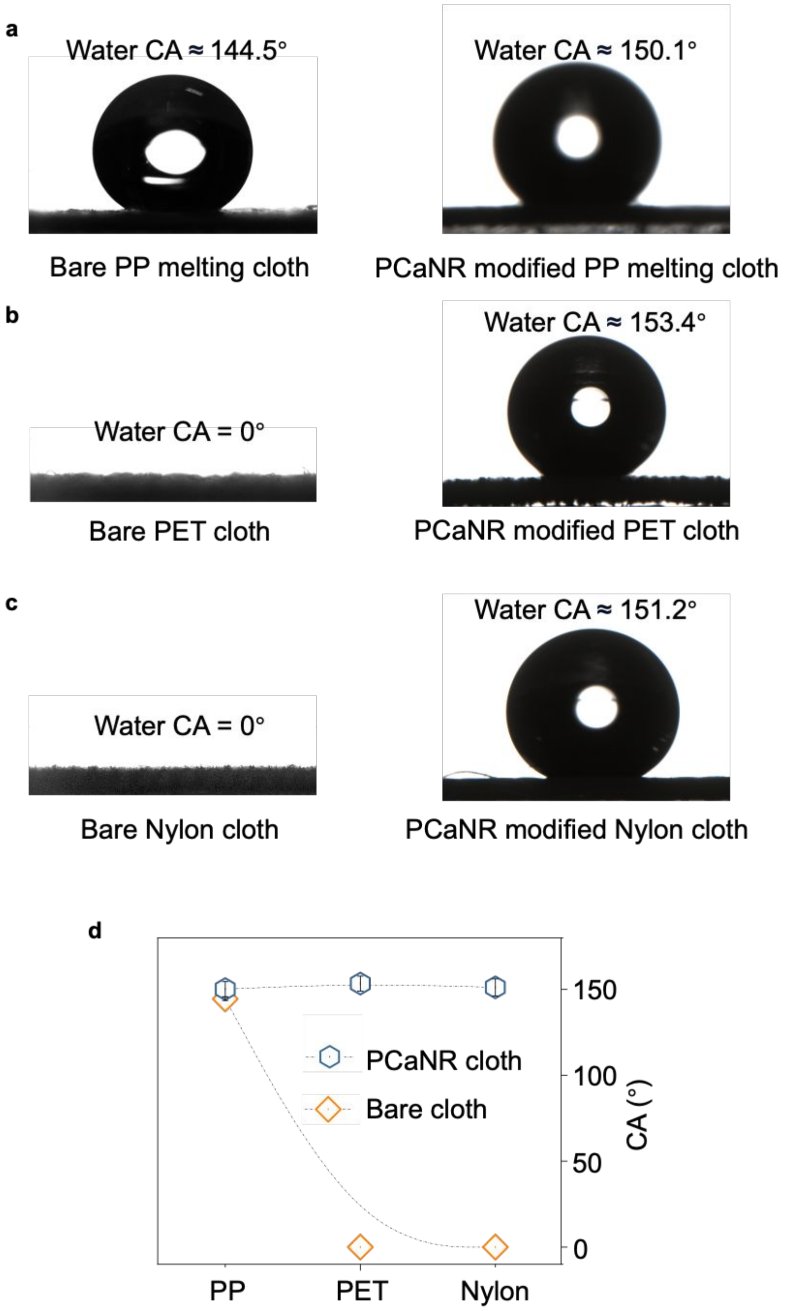

Supplementary Fig. 15 Wettability of three kinds of cloth before and after modified.

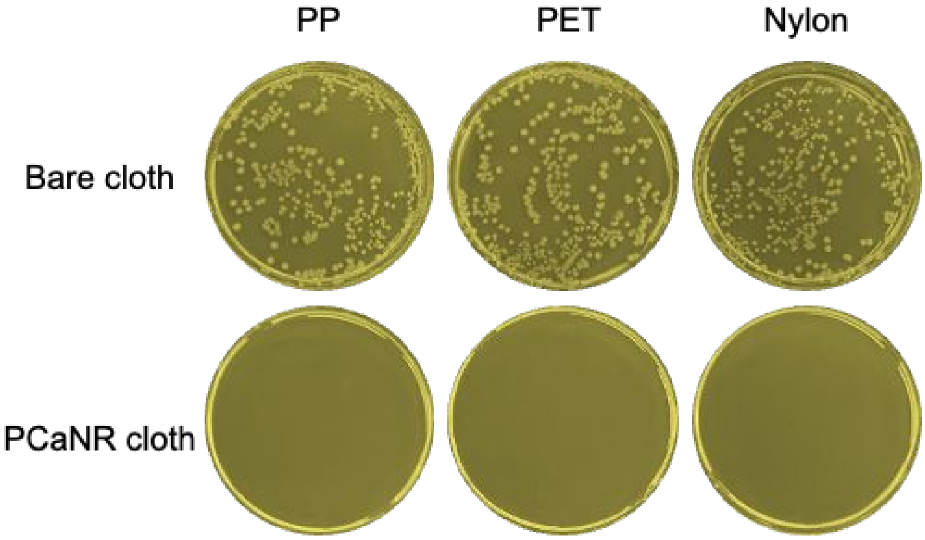

Supplementary Fig. 16 Representative optical images of 3-minute antibacterial assay results of bare cloth and PCaNR based cloth.

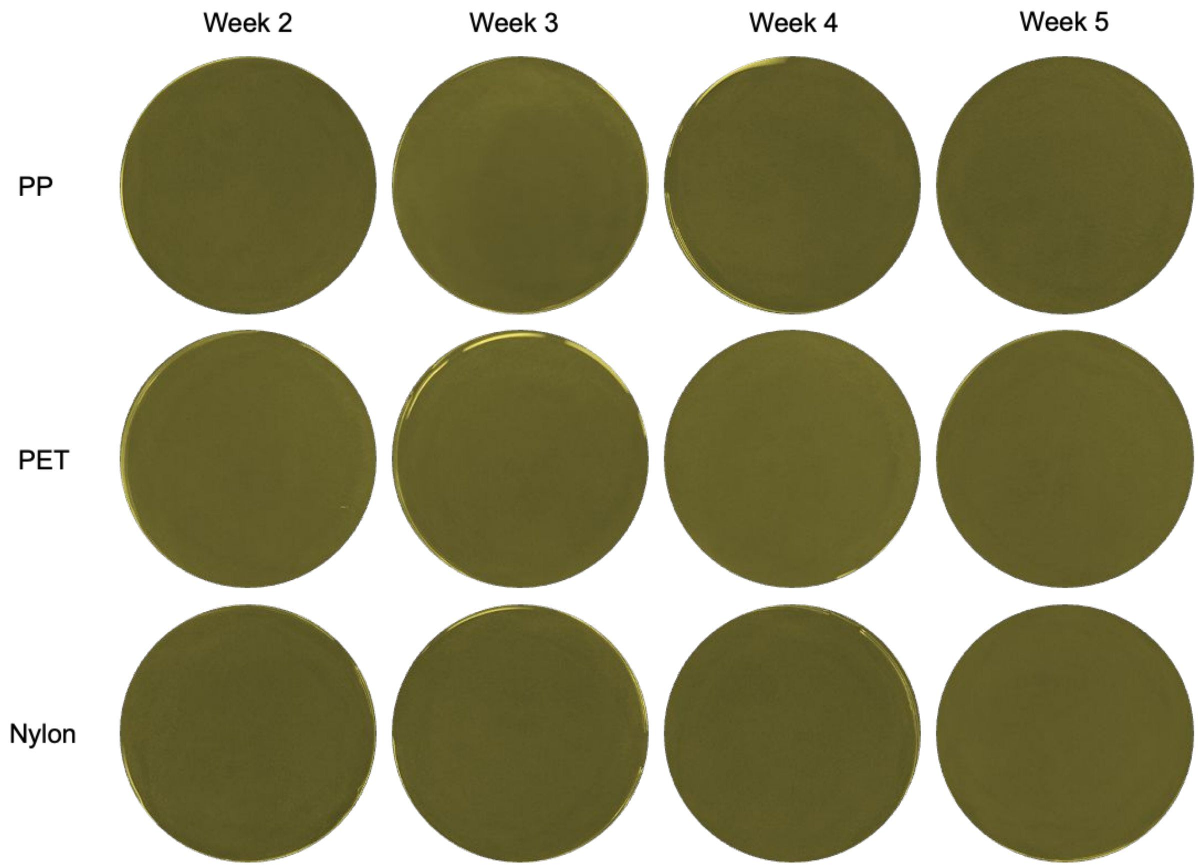

Supplementary Fig. 17 Representative optical images of long-term antibacterial assay results for PCaNR based cloth.

Reference

1. Si, Y. et al. Daylight-driven rechargeable antibacterial and antiviral nanofibrous membranes for bioprotective applications. *Sci. Adv.* **4**, eaar5931 (2018).
2. Li, P. et al. Metal-organic frameworks with photocatalytic bactericidal activity for integrated air cleaning. *Nat. Commun.* **10**, 2177 (2019).
3. Greenspan, L. Humidity fixed-points of binary saturated aqueous-solutions. *J. Res. Natl. Bur. Stand. A. Phys. Chem.* **81**, 89-96 (1977).
4. Huang, Y. et al. Interfacial stresses on droplet interface bilayers using two photon fluorescence lifetime imaging microscopy. *J. Colloid Interface Sci.* **653**, 1196-1204 (2024).
5. Yu, Y. et al. Semi-automated optimization of the CHARMM36 lipid force field to include explicit treatment of long-range dispersion. *J. Chem. Theory Comput.* **17**, 1562-1580 (2021).
6. Jorgensen, W.L., Chandrasekhar, J., Madura, J.D., Impey, R.W. & Klein, M.L. Comparison of simple potential functions for simulating liquid water. *J. Chem. Phys.* **79**, 926-935 (1983).
